# Supplementary material for: Zwitterionic Modification of Polyethyleneimine for Efficient In Vitro siRNA Delivery
Source: Int J Mol Sci. 2022 Apr 30;23(9):5014. doi: 10.3390/ijms23095014 (PMC9100541; doi:10.3390/ijms23095014)
Supplement: Supplementary file 1 [file ijms-23-05014-s001.zip › ijms-1682678-supplementary.pdf]

Supporting Information

Zwitterionic Modification of Polyethyleneimine for Efficient in vitro siRNA Delivery

Fengfan Liu[1], Huahui Su[1], Mengqian Li[1], Wanxuan Xie[1], Yunfeng Yan[2] and Qi Shuai[1]**

[1] National Engineering Research Center for Process Development of Active Pharmaceutical Ingredients, Collaborative Innovation Center of Yangtze River Delta Region Green Pharmaceuticals, Zhejiang University of Technology, Hangzhou, P. R. China

[2] College of Biotechnology and Bioengineering, Zhejiang University of Technology, Hangzhou, Zhejiang 310014, China

* Corresponding author

Qi Shuai - Collaborative Innovation Center of Yangtze River Delta Region Green Pharmaceuticals, Zhejiang University of Technology, Hangzhou 310014, PR China; E-mail: qshuai@zjut.edu.cn

Yunfeng Yan - College of Biotechnology and Bioengineering, Zhejiang University of Technology, Hangzhou, Zhejiang 310014, PR China； E-mail: yfyan@zjut.edu.cn

1. Supporting Data

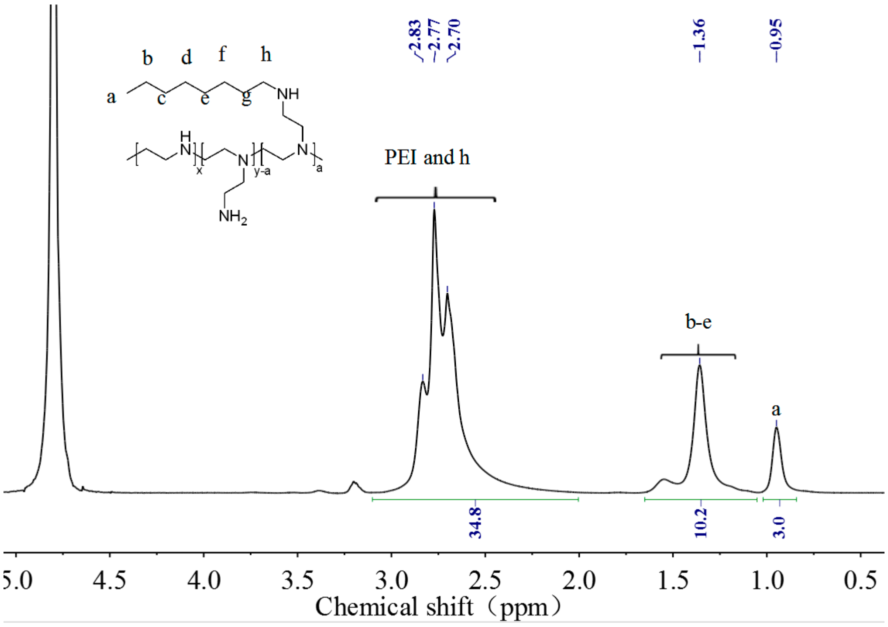

Figure S1. ^{1}H NMR spectra of 25-40

Calculation of the modification ratio of n-octanal to PEI:

$$graft\ ratio = \frac{amount\ of\ octane\ chains\ of\ H-PEI}{amount\ of\ primary\ amine\ groups\ of\ PEI} = \frac{a/3}{PEI/4 * 25\%} = 46.0\%$$

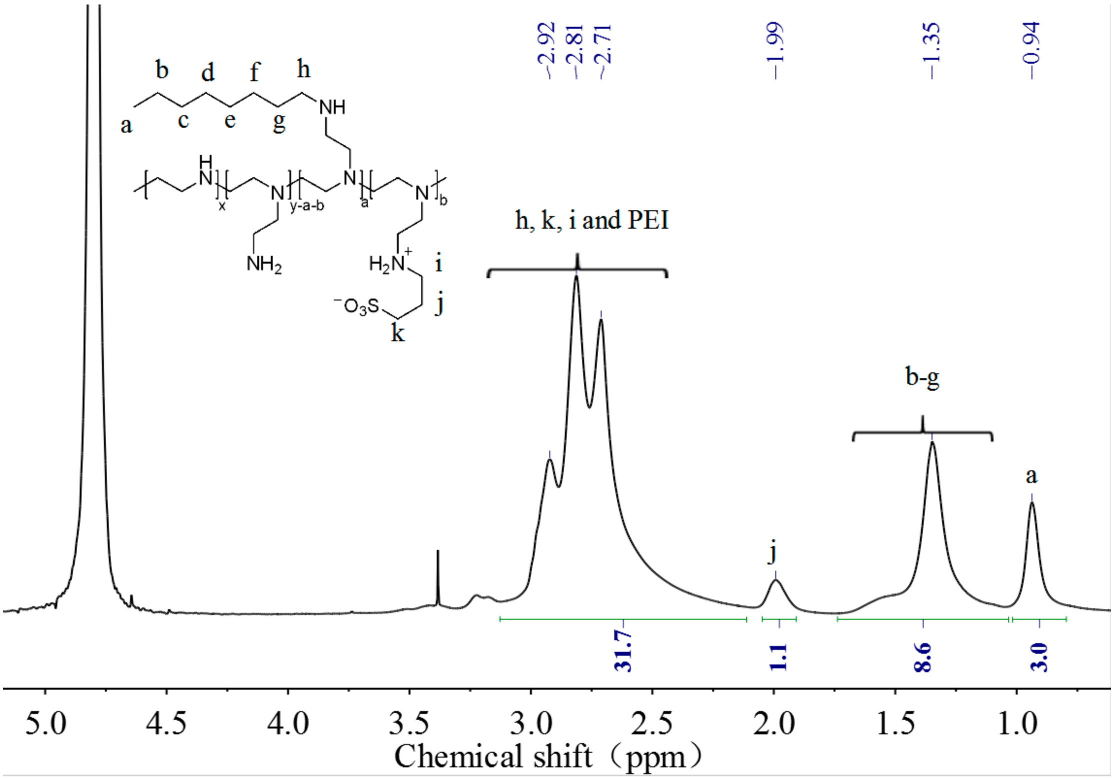

Figure S2. ^{1}H NMR spectra of 25-40-S-20

Calculation of the modification ratio of 1,3 propane sultone to H-PEI:

$$graft\ ratio = \frac{i/2}{a/3} * 40\% = 22\%$$

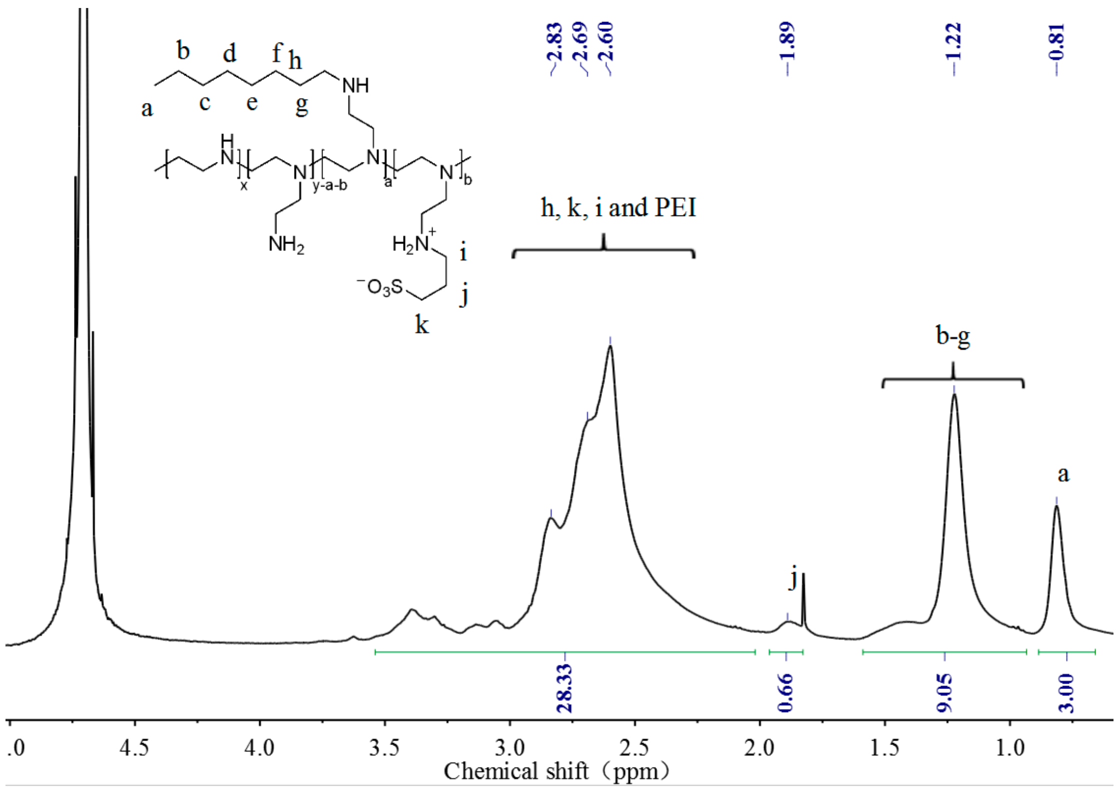

Figure S3. ^{1}H NMR spectra of 25-40-S-10

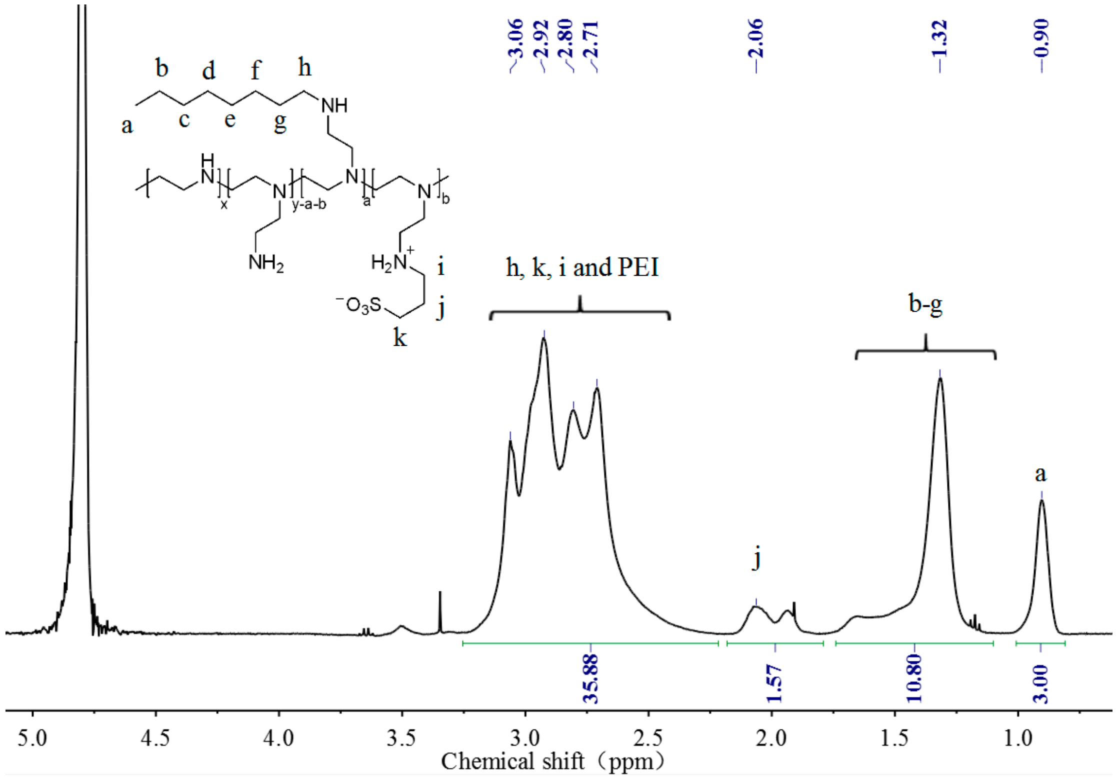

Figure S4. ^{1}H NMR spectra of 25-40-S-30

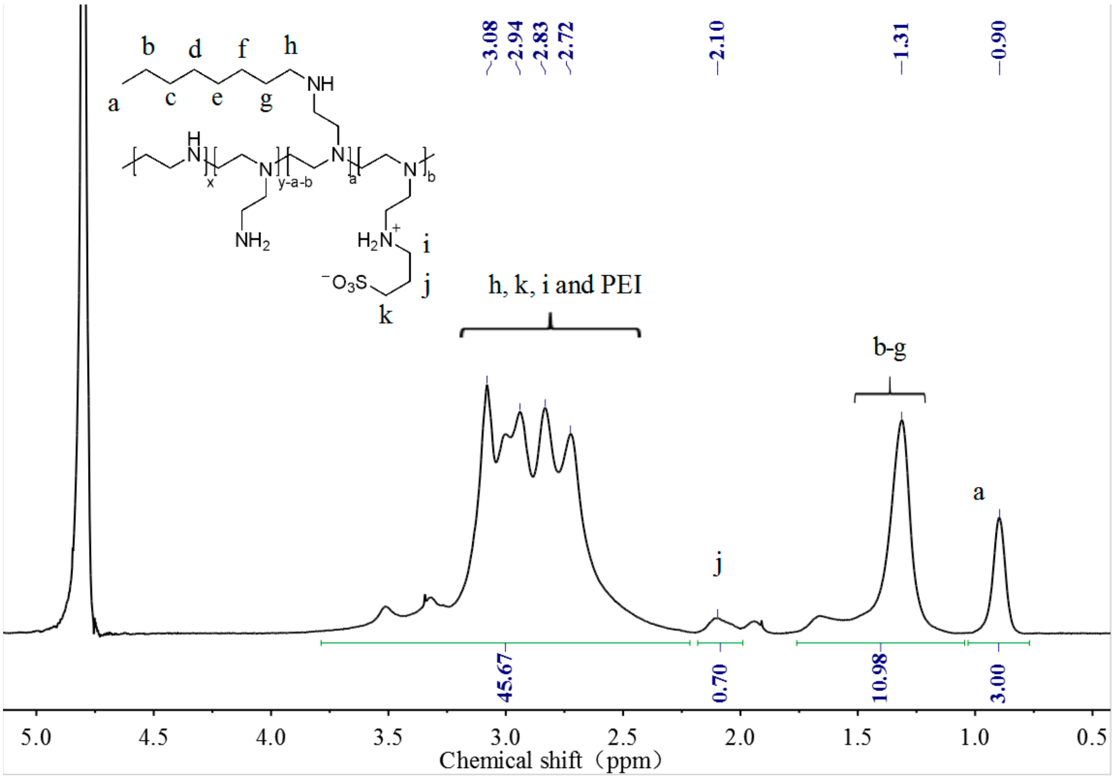

Figure S5. ^{1}H NMR spectra of 10-40-S-10

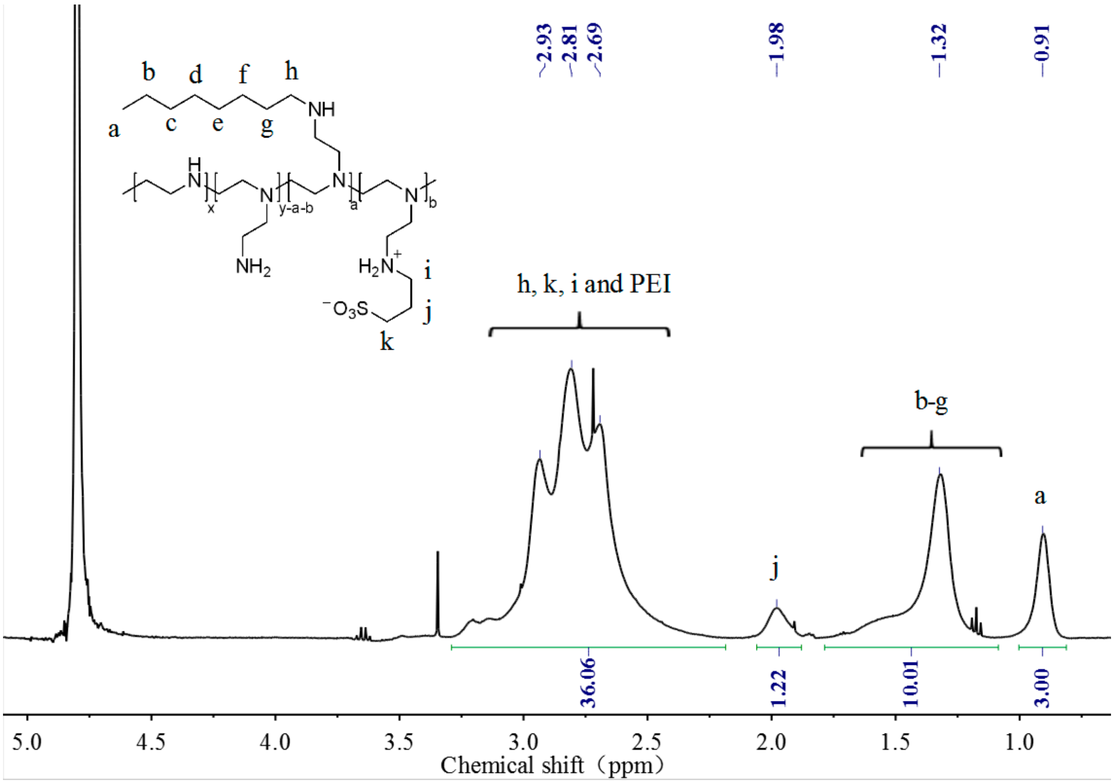

Figure S6. ^{1}H NMR spectra of 10-40-S-20

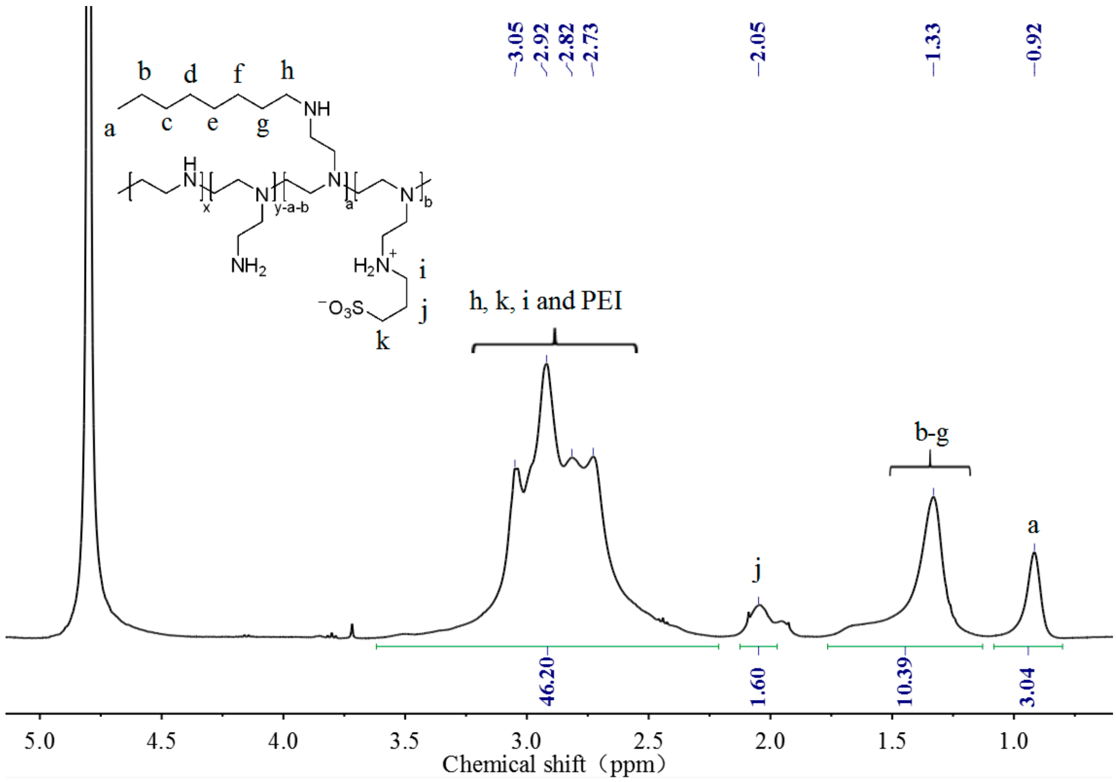

Figure S7. ^1H NMR spectra of 10-40-S-30
